# Prognosis of subtypes of the mucinous breast carcinoma in Chinese women: a population-based study of 32-year experience (1983-2014)

**DOI:** 10.18632/oncotarget.8778

**Published:** 2016-04-18

**Authors:** Bo Pan, Ru Yao, Jie Shi, Qian-Qian Xu, Yi-Dong Zhou, Feng Mao, Yan Lin, Jing-Hong Guan, Xue-Jing Wang, Yan-Na Zhang, Xiao-Hui Zhang, Song-Jie Shen, Ying Zhong, Ya-Li Xu, Qing-Li Zhu, Zhi-Yong Liang, Qiang Sun

**Affiliations:** ^1^ Department of Breast Surgery, Peking Union Medical College Hospital, Chinese Academy of Medical Sciences and Peking Union Medical College, Beijing, P. R. China; ^2^ Department of Pathology, Peking Union Medical College Hospital, Chinese Academy of Medical Sciences and Peking Union Medical College, Beijing, P. R. China; ^3^ Department of Ultrasound, Peking Union Medical College Hospital, Chinese Academy of Medical Sciences and Peking Union Medical College, Beijing, P. R. China

**Keywords:** mucinous breast cancer, subtype, prognosis

## Abstract

**Purpose:**

The heterogeneous nature of the mucinous breast cancer (MBC), with its pure (PMBC) and mixed subtypes (MMBC), calls for precise prognosis assessment.

**Methods:**

We analyzed 197 consecutive MBC patients, including 117 PMBC and 80 MMBC, who were treated from 1983 to 2014. The clinicopathological features, treatment choice, disease-free survival (DFS) and overall survival (OS) were compared among PMBC, MMBC and MMBC subgroups. Prognostic factors of PMBC and MMBC were identified.

**Results:**

Compared to PMBC, MMBC had more lymph node metastasis (p = 0.043), Her2 positivity (*p* = 0.036), high Ki-67 index (defined as>20%, *p* = 0.026) and anti-Her2 targeted therapy (*p* = 0.016). The 5-year DFS of PMBC and MMBC were 90.4% and 86.2%, whereas the 5-year OS were 99.0% and 98.7%. No significant difference was found in DFS or OS among all MBC subtypes. High Ki-67 (*p* = 0.020) appeared as DFS factor in PMBC, while anti-Her2 targeted therapy (*p* = 0.047) as the DFS predictors in MMBC.

**Conclusion:**

MMBC manifested similar 5-year survival to PMBC in Chinese woman, suggesting that intra-tumoral heterogeneity might not interfere with MBC short-term prognosis.

## INTRODUCTION

Synonymous with colloid, gelatinous mucous or mucoid carcinoma, mucinous breast cancer (MBC) represents 1-7% of all breast cancers [1–5]. The World Health Organization designates two subtypes: 1) pure mucinous breast cancer (PMBC) if the non-mucinous component is less than 10% and 2) mixed mucinous breast cancer (MMBC) if there is 10-49% non-mucinous co-existing disease in the tumor [6, 7]. It is generally accepted that PMBC has a favorable prognosis in both Caucasian and Chinese women compared to invasive ductal carcinomas (IDC) [1, 2]. However, most of the studies proposing that MMBC had worse prognosis than PMBC were performed 2-3 decades ago, when modern adjuvant chemotherapy, radiation, endocrine and anti-Her2 targeted therapy were largely unavailable [3, 4, 8–10]. Few studies had investigated the tumor biology, treatment choice and survival outcomes of MMBC in Chinese population, especially with respect to the intra- and inter-tumoral histological heterogeneity represented by the different co-existing cancer components. The prognostic predictors for PMBC and MMBC also remained unclear. A recent study showed that both the mucinous and the co-existing components in MMBC were remarkably similar at the molecular level to PMBC, suggesting that MMBC be best classified as variants of mucinous cancers rather than of IDC [11]. Conversely PMBC appeared to possess phenotypic plasticity that could be converted by estrogen into MMBC with invasive lobular carcinoma (ILC) component [12]. Thus, we plan to compare the prognosis of PMBC *versus* MMBC in Chinese population when all measures of the modern comprehensive therapy were available.

## RESULTS

### Descriptive information of the study cohort

**Figure 1:**
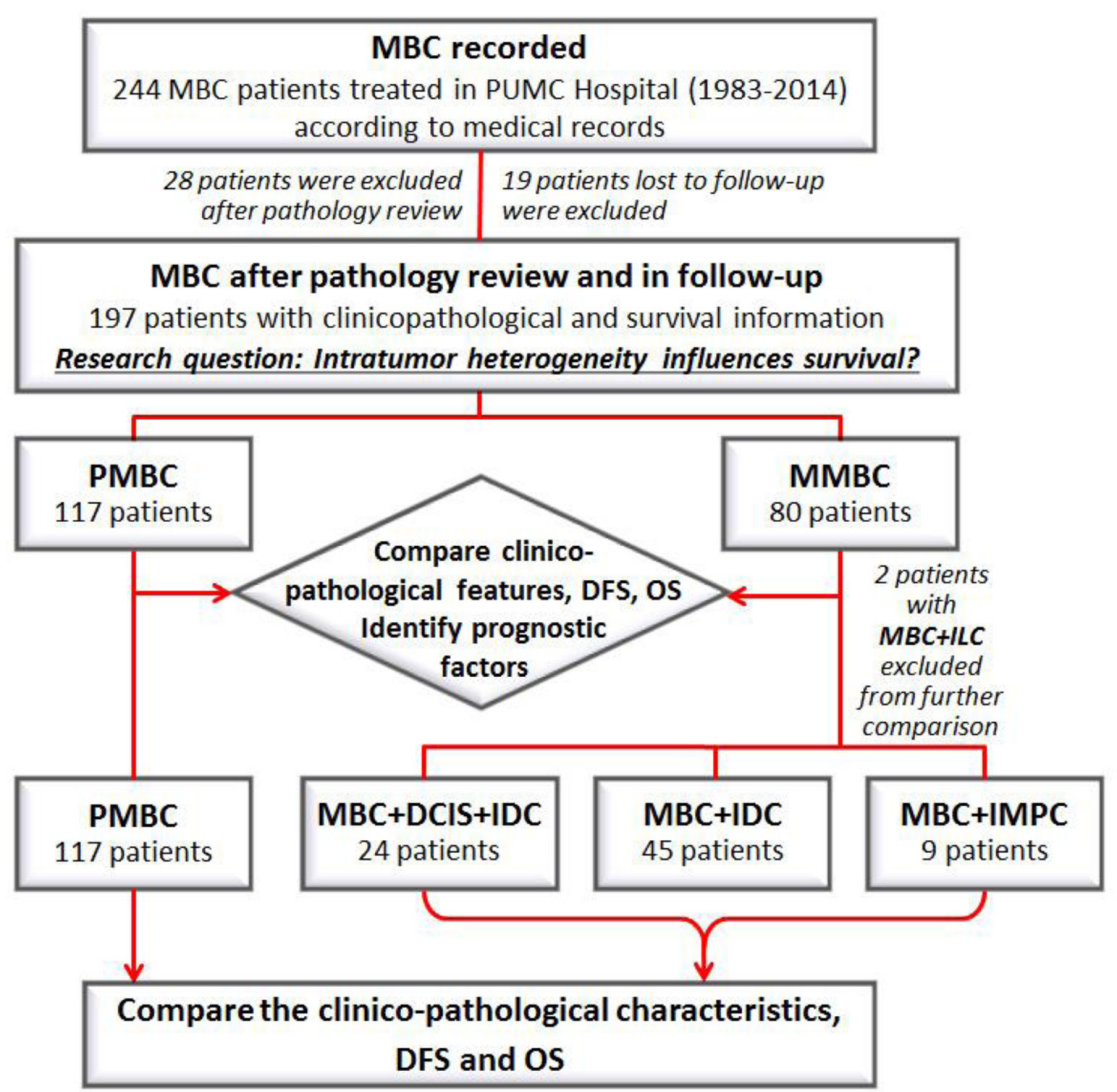
Diagram of the research design The clinic-pathological characteristics and the survival outcomes (DFS and OS) were firstly compared between PMBC and MMBC, and then between PMBC, MBC+DCIS+IDC, MBC+IDC and MBC+IMPC. Two patients with MBC+ILC were excluded from the second comparison due to limited case number. Abbreviations: MBC, mucinous breast cancer; PMBC, pure mucinous breast cancer; MMBC, mixed mucinous breast cancer; DCIS, ductal carcinoma *in situ*; IDC, invasive ductal carcinoma; ILC, invasive lobular carcinoma; IMPC, invasive micropapillary carcinoma; DFS, disease free survival; OS, overall survival.

A total of 244 patients were identified as described in METHOD. After excluding 28 patients with < 50% focal mucinous lesion and 19 patients lost to follow-up, 197 patients were included in the analysis, comprising 1.9% of contemporary 10,192 breast cancer treated in PUMC Hospital. 171 patients (86.8%) were treated during the recent ten years (2005-2014) while 130 patients (66.0%) were treated during the recent five years (2010-2014). 112 patients (56.9%) were pre-menopausal and 85 (43.1%) post-menopausal. There were 117 PMBC and 80 MMBC patients, the latter including 24 patients with ductal carcinoma *in situ* (DCIS) and IDC (with or without other types of carcinoma), 45 with only IDC, 9 with invasive micro-papillary carcinoma (IMPC) and 2 with ILC. With a median follow-up time of 41 months (3-385 months), 11 PMBC and 7 MMBC patients developed recurrence or metastasis, and 1 PMBC and 1 MMBC passed away (Figure 1).

### Comparison of clinicopathological characteristics between subtypes and subgroups of MBC

Compared to PMBC, MMBC had significantly more lymph node metastasis (*p* = 0.043), Her2 positivity (*p* = 0.036), high Ki-67 index (defined as > 20%, *p* = 0.026) and anti-Her2 targeted therapy (*p* = 0.016). There were no significant differences in age at diagnosis, age group distribution, tumor size, TNM stage, ER, PR, hormone receptor status, immunophenotype, p53, type of surgery, chemotherapy, radiotherapy and endocrine therapy (Table 1). When the comparison was performed among PMBC, MBC+DCIS+IDC, MBC+IDC and MBC+IMPC, significant differences were identified in lymph node metastasis (*p* = 0.023), Her2 positivity (*p* = 0.014), high Ki-67 index (*p* = 0.008), chemotherapy (*p* = 0.011) and anti-Her2 targeted therapy (*p* = 0.002) (Table 2).

**Table 1:** Clinicopathological characteristics of PMBC and MMBC patients

Characteristics	No. (%) of Patients		*P*^a^
	PMBC	MMBC	
**Total**	117	80	
**Age (Mean±SD) (years)**	53.26±15.25	55.90±14.38	0.223
**Age at diagnosis (years)**			0.432
≤35	10 (8.5)	5 (6.2)	
36~50	52 (44.5)	30 (37.5)	
>50	55 (47.0)	45 (56.3)	
**Tumor size (cm)**			0.480
T≤2.0	59 (50.4)	43 (53.8)	
2<T≤5.0	49 (41.9)	31 (38.8)	
T>5.0	4 (3.4)	5 (6.2)	
Unknown	5 (4.3)	1 (1.2)	
**Lymph node status**			**0.043**
Negative	103 (88.0)	64 (80.0)	
Positive	11 (9.4)	16 (20.0)	
Unknown	3 (2.6)	0 (0.0)	
**TNM stage**^b^			0.147
Stage I	55 (47.0)	35 (43.8)	
Stage II	53 (45.3)	35 (43.8)	
Stage III	6 (5.1)	10 (12.5)	
Unknown	3 (2.6)	0 (0.0)	
**ER status**			0.484
Positive	94 (80.3)	66 (82.5)	
Negative	12 (10.3)	10 (12.5)	
Unknwon	11 (9.4)	4 (5.0)	
**PR status**			0.834
Positive	88 (75.3)	62 (77.6)	
Negative	19 (16.2)	13 (16.2)	
Unknwon	10 (8.5)	5 (6.2)	
**Hormone receptor status**			0.631
Positive	98 (83.8)	70 (87.5)	
Negative	9 (7.7)	6 (7.5)	
Unknwon	10 (8.5)	4 (5.0)	
**HER2 status**			**0.036**
Positive	3 (2.6)	9 (11.2)	
Negative	97 (82.9)	58 (72.5)	
Unknwon	17 (14.5)	13 (16.2)	
**Ki-67 expression**			**0.026**
<20%	64 (54.7)	36 (45.0)	
≥20%	29 (24.8)	34 (42.5)	
Unknown	24 (20.5)	10 (12.5)	
**Immunophenotype**			0.136
Luminal A	62 (53.0)	32 (40.0)	
Luminal B	29 (24.8)	32 (40.0)	
HER2	0 (0.0)	1 (1.2)	
TNBC	8 (6.8)	5 (6.2)	
Unknown	18 (15.4)	10 (12.5)	
**p53**			0.547
Positive	6 (5.1)	6 (7.5)	
Negative	46 (39.3)	26 (32.5)	
Unknown	65 (55.6)	48 (60.0)	
**Surgery**			0.897
Mastectomy	75 (64.1)	52 (65.0)	
Breast conserving	42 (35.9)	28 (35.0)	
Unknown	0 (0.0)	0 (0.0)	
**Chemotherapy**			0.195
No	69 (59.0)	41 (51.2)	
Yes	43 (36.8)	38 (47.5)	
Unknown	5 (4.2)	1 (1.2)	
**Radiotherapy**			0.242
No	82 (70.1)	57 (71.3)	
Yes	26 (22.2)	21 (20.2)	
Unknown	9 (7.7)	2 (2.5)	
**Anti-Her2 targeted therapy**			**0.016**
No	107 (91.5)	70 (87.5)	
Yes	2 (1.7)	8 (10.0)	
Unknown	8 (6.8)	2 (2.5)	
**Endocrine therapy**			0.399
No	15 (12.8)	8 (10.0)	
Yes	91 (77.8)	68 (85.0)	
Unknown	11 (9.4)	4 (5.0)	

Abbreviations: PMBC, pure mucinous breast cancer; MBC, mucinous breast cancer; SD, standard deviation; TNM, tumor, node, metastasis system; ER, estrogen receptor; PR, progesterone receptor; TNBC, triple negative breast cancer.

a Bold type indicates statistical significance.

b TNM stage is according to the 7^th^ AJCC cancer staging system.

**Table 2:** Comparison of the clinicopathological characteristics of PMBC versus subgroups of MMBC including MBC+DCIS+IDC, MBC+IDC and MBC+IMPC patients

Characteristics	No. (%) of Patients				*P*^a^
	PMBC	MBC+DCIS+IDC	MBC+IDC	MBC+IMPC	
**Total**	117	24	45	9	
**Age (Mean±SD) (years)**	53.3±15.3	56.6±12.6	54.7±15.2	61.8±15.4	0.333
**Age at diagnosis (years)**					0.612
≤35	10 (8.5)	0 (0.0)	4 (8.9)	1 (11.1)	
36~50	52 (44.5)	10 (41.7)	17 (37.8)	2 (22.2)	
>50	55 (47.0)	14 (58.3)	24 (53.3)	6 (66.7)	
**Tumor size (cm)**					0.204
T≤2.0	59 (50.4)	18 (75.0)	19 (42.2)	5 (56.6)	
2<T≤5.0	49 (41.9)	4 (16.7)	24 (53.3)	3 (33.3)	
T>5.0	4 (3.4)	1 (4.2)	2 (4.4)	1 (11.1)	
Unknown	5 (4.3)	1 (4.2)	0 (0.0)	0 (0.0)	
**Lymph node status**					**0.023**
Negative	103 (88.0)	22 (91.7)	32 (71.1)	9 (100.0)	
Positive	11 (9.4)	2 (8.3)	13 (28.9)	0 (0.0)	
Unknown	3 (2.6)	0 (0.0)	0 (0.0)	0 (0.0)	
TNM stage^b^					0.069
Stage I	55 (47.0)	16 (66.7)	13 (28.9)	5 (55.6)	
Stage II	53 (45.3)	6 (25.0)	25 (55.6)	4 (44.4)	
Stage III	6 (5.1)	2 (8.3)	7 (15.6)	0 (0.0)	
Unknown	3 (2.6)	0 (0.0)	0 (0.0)	0 (0.0)	
**ER status**					0.587
Positive	94 (80.3)	22 (91.7)	34 (75.6)	8 (88.9)	
Negative	12 (10.3)	2 (8.3)	7 (15.6)	1 (11.1)	
Unknown	11 (9.4)	0 (0.0)	4 (8.9)	0 (0.0)	
**PR status**					0.816
Positive	88 (75.2)	21 (87.5)	33 (73.3)	7 (77.8)	
Negative	19 (16.2)	2 (8.3)	8 (17.8)	2 (22.2)	
Unknown	10 (8.5)	1 (4.2)	4 (8.9)	0 (0.0)	
**Hormone receptor status**					0.694
Positive	98 (83.8)	23 (95.8)	37 (82.2)	8 (88.9)	
Negative	9 (7.7)	1 (4.2)	4 (8.9)	1 (11.1)	
Unknown	10 (8.5)	0 (0.0)	4 (8.9)	0 (0.0)	
**HER2 status**					**0.014**
Positive	3 (2.6)	1 (4.2)	8 (17.8)	0 (0.0)	
Negative	97 (82.9)	20 (83.3)	28 (62.2)	8 (88.9)	
Unknown	17 (14.5)	3 (12.5)	9 (20.0)	1 (11.1)	
**Ki-67 expression**					**0.008**
<20%	64 (54.7)	16 (66.7)	14 (31.1)	6 (66.7)	
≥20%	29 (24.8)	5 (20.8)	24 (53.3)	3 (33.3)	
Unknown	24 (20.5)	3 (12.5)	7 (15.6)	0 (0.0)	
**Immunophenotype**					0.105
Luminal A	62 (53.0)	15 (62.5)	12 (26.7)	5 (55.6)	
Luminal B	29 (24.8)	5 (20.8)	22 (48.9)	3 (33.3)	
HER2	0 (0.0)	0 (0.0)	1 (2.2)	0 (0.0)	
TNBC	8 (6.8)	1 (4.2)	3 (6.7)	1 (11.1)	
Unknown	18 (15.4)	3 (12.5)	7 (15.6)	0 (0.0)	
**p53**					0.418
Positive	6 (5.1)	0 (0.0)	5 (11.1)	1 (11.1)	
Negative	46 (39.3)	7 (29.2)	16 (35.6)	2 (22.2)	
Unknown	65 (55.6)	17 (70.8)	24 (53.3)	6 (66.7)	
**Surgery**					0.575
Mastectomy	75 (64.1)	15 (62.5)	31 (68.9)	4 (44.4)	
Breast conserving	42 (35.9)	9 (37.5)	14 (31.1)	5 (55.6)	
Unknown	0 (0.0)	0 (0.0)	0 (0.0)	0 (0.0)	
**Chemotherapy**					**0.011**
No	69 (59.0)	18 (75.0)	16 (35.6)	6 (66.7)	
Yes	43 (36.8)	5 (20.8)	29 (64.4)	3 (33.3)	
Unknown	5 (4.2)	1 (4.2)	0 (0.0)	0 (0.0)	
**Radiotherapy**					0.299
No	82 (70.1)	19 (79.2)	30 (66.7)	6 (66.7)	
Yes	26 (22.2)	4 (16.7)	15 (33.3)	3 (33.3)	
Unknown	9 (7.7)	1 (4.1)	0 (0.0)	0 (0.0)	
**Anti-Her2 targeted therapy**					**0.002**
No	107 (91.5)	23 (95.8)	36 (80.0)	9 (100.0)	
Yes	2 (1.7)	0 (0.0)	8 (17.8)	0 (0.0)	
Unknown	8 (6.8)	1 (4.1)	1 (2.2)	0 (0.0)	
**Endocrine therapy**					0.566
No	15 (12.8)	1 (4.2)	6 (13.3)	1 (11.1)	
Yes	91 (77.8)	21 (87.5)	38 (84.4)	8 (88.9)	
Unknown	11 (9.4)	2 (8.3)	1 (2.2)	0 (0.0)	

Abbreviations: PMBC, pure mucinous breast cancer; MBC, mucinous breast cancer; DCIS, ductal carcinoma in situ; IDC, invasive ductal carcinoma; IMPC, invasive micropapillary carcinoma; SD, standard deviation; TNM, tumor, node, metastasis system; ER, estrogen receptor; PR, progesterone receptor.

a Bold type indicates statistical significance.

b TNM stage is according to the 7^th^ AJCC cancer staging system.

**Figure 2:**
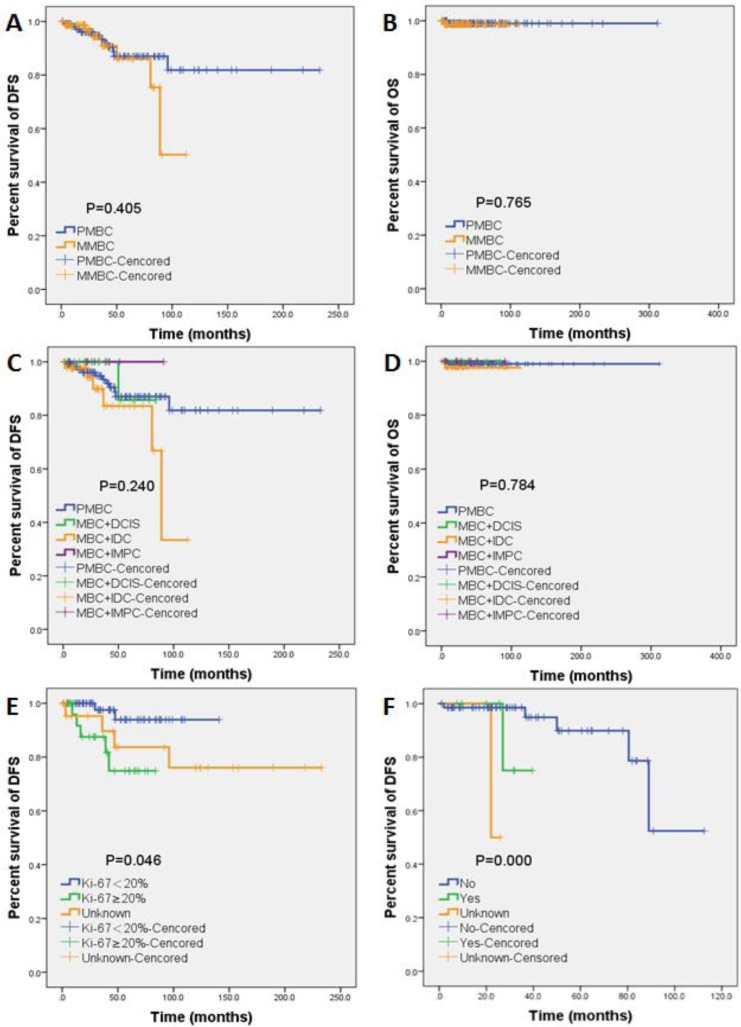
Kaplan-Meier estimates of DFS and OS of MBC patients DFS **A.** and OS **B.** compared between PMBC and MMBC patients. The comparison of DFS **C.** and OS **D.** of PMBC, MBC+DCIS+IDC, MBC+IDC, and MBC+IMPC patients. DFS of PMBC patients compared between subgroups of Ki-67 high (defined as ≥ 20%) *versus* Ki-67 low (defined as < 20%) **E.** DFS of MMBC patients compared between patient subgroups with or without anti-Her2 targeted therapy **F.**.

### Survival outcomes and prognostic factors of MBC subtypes

The 5-year DFS of PMBC and MMBC were 90.4% and 86.2%, whereas the 5-year OS were 99.0% and 98.7% respectively. The 5-year DFS and OS for MMBC subgroups were: 85.7% and 100.0% for MBC+DCIS+IDC, 83.5% and 97.6% for MBC+IDC, and 100.0% and 100.0% for MBC+IMPC. No significant difference was found in DFS or OS either between PMBC *vs* MMBC or among the above mentioned MMBC subgroups (Figure 2, Tables 3, 4). High Ki-67 index (defined as > 20%, *p* = 0.020) was identified as the significant DFS prognostic factor for PMBC, whereas anti-Her2 targeted therapy (*p* = 0.047) appeared to be the DFS predictor for MMBC (Tables 5, 6). DFS stratified by Ki-67 in PMBC and by anti-Her2 targeted therapy in MMBC both showed significant differences (Figure 2). ER, PR, hormone receptor status, immunophenotype and endocrine therapy might be potential DFS predictors according to univariate analysis. However, these factors were not significant in the multivariate analysis. None of the clinicopathological and treatment factors listed above could serve as OS predictors for MBC subtypes due to the limited OS events.

**Table 3:** Kaplan-Meier estimated DFS and OS rates compared between PMBC and MMBC

Group	No. of patients	5-year DFS (%)	P	5-year OS (%)	P
PMBC	117	90.4	0.405	99.0	0.765
MMBC	80	86.2		98.7	

Abbreviations: PMBC, pure mucinous breast cancer; MMBC, mixed mucinous breast cancer; DFS, disease free survival; OS, overall survival.

**Table 4:** Kaplan-Meier estimated DFS and OS rates compared between PMBC, MBC+DCIS+IDC, MBC+IDC and MBC+IMPC

Group	No. of patients	5-year DFS (%)	*P*	5-year OS (%)	*P*
PMBC	117	90.4	0.240	99.0	0.784
MBC+DCIS+IDC	24	85.7		100.0	
MBC+IDC	45	83.5		97.6	
MBC+IMPC	9	100.0		100.0	

Abbreviations: MBC, mucinous breast cancer; PMBC, pure mucinous breast cancer; MMBC, mixed mucinous breast cancer; DCIS, ductal carcinoma in situ; IDC, invasive ductal carcinoma; IMPC, invasive micropapillary carcinoma; DFS, disease free survival; OS, overall survival.

**Table 5:** Univariate and multivariate Cox analysis of disease-free survival of patients with PMBC

Variables	Univariate^a^	Multivariate^b^	
	*P*^c^	HR (95% CI)	*P*^c^
**Age at diagnosis**	0.170	0.258 (0.056-1.186)	0.082
**Tumor size**	0.415	0.358 (0.023-5.662)	0.466
**Lymph node status**	0.331	5.666 (0.358-89.609)	0.218
**TNM staged**	0.831	2.546 (0.083-78.191)	0.593
**ER status**	0.697	1512.053 (0-9.453E+138)	0.963
**PR status**	0.496	0.247 (0.028-2.203)	0.210
**Hormone receptor status**	0.741	0.004 (0-2.834E+133)	0.973
**HER2 status**	0.631	1.796 (0.359-8.988)	0.476
**Ki-67 expression**	**0.046**	**58.722 (1.889-1825.766)**	**0.020**
**Immunophenotype**	0.111	0.169 (0.025-1.135)	0.067
**p53**	0.801	1.857 (0.676-5.106)	0.230
**Surgery**	0.054	0.077 (0.005-1.227)	0.070
**Chemotherapy**	0.379	0.420 (0.070-2.517)	0.082
**Radiotherapy**	0.738	1.051 (0.095-11.576)	0.070
**Anti-Her2 targeted therapy**	0.874	0.078 (0.004-1.629)	0.466
**Endocrine therapy**	0.945	8.401 (0.402-175.734)	0.218

Abbreviations: PMBC, pure mucinous breast cancer; ER, estrogen receptor; PR, progesterone receptor.

a Kaplan-Meier univariate analysis including all factors.

b Adjusted by Cox proportional hazard regression model including all factors with method of enter.

c Bold type indicates statistical significance.

d TNM stage is according to the 7^th^ AJCC cancer staging system.

**Table 6:** Univariate and multivariate Cox analysis of disease-free survival of patients with MMBC

Variables	Univariate^a^	Multivariate^b^	
	*P*^c^	HR (95% CI)	*P*^c^
**Pathologic types**	0.460	0.344 (0.015-8.149)	0.509
**Age at diagnosis**	0.606	1.083 (0.048-24.428	0.960
**Tumor size**	0.764	0.052 (0.000-31.232)	0.364
**Lymph node status**	0.573	0.000 (0.000-355.483)	0.145
**TNM staged**	0.618	3154 (0.032-3126)	0.170
**ER status**	**0.000**	0.004 (0.000-5.52)	0.136
**PR status**	**0.005**	3.696 (0.039-347.957)	0.573
**Hormone receptor status**	**0.000**	3.246 (0.004-2777)	0.733
**HER2 status**	0.504	0.092 (0.002-3.521)	0.199
**Ki-67 expression**	0.302	12.349 (0.005-33822)	0.534
**Immunophenotype**	**0.000**	1.055 (0.055-20.270)	0.971
**p53**	0.067	2.025 (0.086-47.626)	0.662
**Surgery**	0.962	0.025 (0.000-176.301)	0.414
**Chemotherapy**	0.232	0.172 (0.001-23.738)	0.483
**Radiotherapy**	0.473	27.030 (0.012-60963)	0.403
**Anti-Her2 targeted therapy**	**0.000**	**6977 (1.1410-42696079)**	**0.047**
**Endocrine therapy**	**0.003**	0.071 (0.001-3.380)	0.180

Abbreviations: MMBC, mixed mucinous breast cancer; ER, estrogen receptor; PR, progesterone receptor.

a Kaplan-Meier univariate analysis including all factors.

b Adjusted by Cox proportional hazard regression model including all factors with method of enter.

c Bold type indicates statistical significance.

d TNM stage is according to the 7^th^ AJCC cancer staging system.

## DISCUSSION

MBC is one of the most commonly seen special types of breast cancer [1, 2, 4, 8]. Experience in diagnosis and treatment of MBC was usually acquired from retrospective studies instead of prospective randomized trials. It was widely believed that MMBC had a poorer prognosis than PMBC [3, 4, 8–10]. However, these retrospective studies were mainly based on data from Caucasian, and mostly performed during the 1960s to 1980s, when anti-Her2 targeted therapy, most of the endocrine therapy, chemotherapy and radiation therapy were unavailable. Thus the poorer outcome of MMBC might be due to insufficient treatment. Additionally, MMBC is not a single disease. Whether MMBC subgroups have different survival outcomes remains unclear. A recent study reported differences in breast cancer epidemiology, clinical characteristics and prognosis between Chinese and Caucasian women [13, 14]. However, few studies have evaluated the survival outcome among MBC subtypes in Chinese women, who tend to develop breast cancer and MBC at a much younger age than Caucasian counterparts [1, 2, 5, 15, 16].

Although PMBC usually had normal diploid DNA stemline whereas MMBC harbored aneuploid DNA content, a recent study suggested that MBC subtypes based on gene expression profiling might be more complex than anticipated [17, 18]. Unsupervised clustering analysis showed that MMBC displayed similar patterns of genetic aberrations and preferentially clustered together with PMBC rather than with IDC [11]. A study with MBC cell line and xenograft model also showed that PMBC manifested phenotypic plasticity and could be converted by estrogen into MMBC with ILC [12]. This genotypic and phenotypic similarity between PMBC and MMBC provides explanation for their similar prognosis. Secretory mucins (MUC2 and MUC6) and the mucus might also act as a barrier to cancerous extension and decrease the aggressiveness of the tumor biology [8, 19].

In our study, the difference in lymph node (LN) metastasis between PMBC, MMBC and MMBC subtypes might be due to distinct tumor biology. However, the MBC were diagnosed at similar T stage and hence have no significant differences in pTNM stage. Our result on MBC survival coincided with the study from Park S et al. reporting similar 10-year DFS and OS between PMBC and MMBC [20]. Bae SY et al. reported similar DFS and different OS (*p* = 0.043), however, their study did not review pTNM stage, Ki-67, anti-Her2 targeted therapy [21]. Additionally, the age at diagnosis of MBC patients was much younger than contemporary IDC in Korean women, which was different from both Caucasian and Chinese [1, 2, 5, 20, 21]. Zhang M et al. reported better PMBC survival than MMBC in Chinese population [5]. However, most of the MMBC patients included in that study were diagnosed at a much later stage than PMBC, while 70.5% of PMBC patients received chemotherapy. There was no data concerning the Her2 status so that the similar percentage of anti-Her2 targeted therapy between MBC *vs* non-MBC or between PMBC *vs* MMBC would be difficult to interpret.

Compared with IDC, IMPC usually has larger size, more metastatic lymph nodes, increased lymphovascular invasion (LVI) and more aggressive behavior [22]. Poorer survival was also observed for breast carcinoma containing IMPC component [22]. Notably, a special subtype of PMBC with micropapillary epithelial growth pattern was identified as invasive micropapillary mucinous carcinoma (IMPMC) [23], or mucinous carcinomas with a micropapillary pattern (MUMPC) [24]. This heterogeneous PMBC had more LN metastasis, higher Her2 expression, LVI, and a poorer prognosis than pure PMBC [23, 24]. Meanwhile, it showed decreased LN metastasis, lower nuclear grade, higher expression of ER and PR, less expression of Her2, and better prognosis compared to IMPC. Though controversial, it was proposed that PMBC, MUMPC/IMPMC and IMPC might represent clinical entities within a spectrum of heterogeneous diseases, with different percentage of mucin secretion and micropapillary components [23–25]. The MBC+IMPC in our study was different from MUMPC/IMPMC and did not exhibit higher LN metastasis, higher Her2 or Ki-67 expression, or poorer survival outcome.

Our study had several limitations. Firstly, it was a single-center study with limited case number, and two patients with MBC+ILC had to be excluded from the comparison. Secondly, although this retrospective study reviewed MBC patients distributed during 32 years' time span, majority (86.8%) of patients was treated in the recent decade (2005-2014), so it would make more sense to analyze the 5-year short-term survival. There might still be significant difference in long-term 10-year prognosis between PMBC and MMBC, because MBC is basically luminal subtype and have shown late recurrences after 10 years [24, 26]. Thirdly, LN metastasis was not identified as the DFS predictor in our study, although it was identified in other studies to be candidate prognostic factor for PMBC [1, 2, 8, 21, 26, 27]. Fourthly, Ki-67 expression was only documented in 79.5% of the PMBC and 87.5% of the MMBC, while p53 status in more than half of the cases was unknown.

In conclusion, our study revealed that MMBC had similar short-term survival as PMBC in Chinese patients, suggesting that intra-tumoral heterogeneity might not interfere with MBC prognosis in Chinese woman. Ki-67 proliferation index was identified as a DFS prognostic factor for PMBC, whereas anti-Her2 targeted therapy as the potential DFS predictor for MMBC. Further studies with increased cases number, prolonged follow-up and improved bio-markers need to be performed to gain a deeper understanding of MBC biology and prognosis with respect to intra- and inter-tumoral heterogeneity.

## MATERIALS AND METHODS

### Ethics statement

This study was approved by the Ethics Committee of the Peking Union Medical College Hospital, Chinese Academy of Medical Sciences.

### Patient selection, pathology review and follow-up

From January 1983 to December 2014, 244 consecutive MBC patients were treated primarily with breast cancer surgeries in PUMC Hospital according to the medical records searching. All patients' formalin-fixed paraffin-embedded (FFPE) pathological sections were reviewed and 28 patients with focal mucinous components < 50% of the total cancerous lesions were excluded from the study. All patients were followed by telephone call, by out-patient clinics records of follow-up examinations or by both measures. Another 19 patients who were lost to follow-up were also excluded.

There were 197 MBC patients, including 117 PMBC and 80 MMBC, in the study cohort. The clinicopathological characteristics, treatment choice, DFS and OS were compared both between 117 PMBC *vs* 80 MMBC, and among all MBC subgroups, including 24 MMBC with DCIS and IDC (with or without other types of carcinoma), 45 with only IDC and 9 with IMPC. Two patients with MMBC and ILC were excluded from the comparison due to the small case number. DFS factors of PMBC and MMBC were identified respectively. Identification of prognostic factors for MMBC subgroups were not performed also due to the limited case numbers (Figure 1).

### Statistical analysis

The quantitative variables were compared with *t*-test and the categorical variables were compared with chi-square tests. Survival outcomes including 5-year predicted DFS and OS were analyzed and compared by the Kaplan-Meier curve method. Kaplan-Meier univariate analyses and Cox multivariate analyses were performed to identify the prognostic factors for PMBC and MMBC respectively. The significance threshold was set at *p* < 0.05. SPSS software, version 18.0 (SPSS, Inc. Chicago, IL, US) was used for all of the statistical analyses.

## References

[1] Di Saverio S, Gutierrez J, Avisar E (2008). A retrospective review with long term follow up of 11,400 cases of pure mucinous breast carcinoma. Breast cancer research and treatment.

[2] Cao AY, He M, Liu ZB, Di GH, Wu J, Lu JS, Liu GY, Shen ZZ, Shao ZM (2012). Outcome of pure mucinous breast carcinoma compared to infiltrating ductal carcinoma: a population-based study from China. Annals of surgical oncology.

[3] Toikkanen S, Kujari H (1989). Pure and mixed mucinous carcinomas of the breast: a clinicopathologic analysis of 61 cases with long-term follow-up. Human pathology.

[4] Andre S, Cunha F, Bernardo M, Meneses e Sousa J, Cortez F, Soares J (1995). Mucinous carcinoma of the breast: a pathologic study of 82 cases. Journal of surgical oncology.

[5] Zhang M, Teng XD, Guo XX, Zhao JS, Li ZG (2014). Clinicopathological characteristics and prognosis of mucinous breast carcinoma. Journal of cancer research and clinical oncology.

[6] Tavassoli FA DP (2003). Pathology and genetics of tumours of the breast and female genital organs. World Health Organization Classification of Tumours.

[7] Lakhani SR, Ellis LO, Schnitt SJ, Tan PH, Van de Vijver MJ (2012). WHO Classification of Tumours of the Breast.

[8] Komaki K, Sakamoto G, Sugano H, Morimoto T, Monden Y (1988). Mucinous carcinoma of the breast in Japan. A prognostic analysis based on morphologic features. Cancer.

[9] Rasmussen BB, Rose C, Christensen IB (1987). Prognostic factors in primary mucinous breast carcinoma. American journal of clinical pathology.

[10] Norris HJ, Taylor HB (1965). Prognosis Of Mucinous (Gelatinous) Carcinoma Of the Breast. Cancer.

[11] Lacroix-Triki M, Suarez PH, MacKay A, Lambros MB, Natrajan R, Savage K, Geyer FC, Weigelt B, Ashworth A, Reis-Filho JS (2010). Mucinous carcinoma of the breast is genomically distinct from invasive ductal carcinomas of no special type. The Journal of pathology.

[12] Jambal P, Badtke MM, Harrell JC, Borges VF, Post MD, Sollender GE, Spillman MA, Horwitz KB, Jacobsen BM (2013). Estrogen switches pure mucinous breast cancer to invasive lobular carcinoma with mucinous features. Breast cancer research and treatment.

[13] Chen DN, Song CG, Ouyang QW, Jiang YZ, Ye FG, Ma FJ, Luo RC, Shao ZM (2015). Differences in breast cancer characteristics and outcomes between Caucasian and Chinese women in the US. Oncotarget.

[14] Fan L, Strasser-Weippl K, Li JJ, St Louis J, Finkelstein DM, Yu KD, Chen WQ, Shao ZM, Goss PE (2014). Breast cancer in China. The Lancet Oncology.

[15] Li J, Zhang BN, Fan JH, Pang Y, Zhang P, Wang SL, Zheng S, Zhang B, Yang HJ, Xie XM, Tang ZH, Li H, Li JY, He JJ, Qiao YL (2011). A nation-wide multicenter 10-year (1999-2008) retrospective clinical epidemiological study of female breast cancer in China. BMC cancer.

[16] Zhang L, Jia N, Han L, Yang L, Xu W, Chen W (2015). Comparative analysis of imaging and pathology features of mucinous carcinoma of the breast. Clinical breast cancer.

[17] Weigelt B, Horlings HM, Kreike B, Hayes MM, Hauptmann M, Wessels LF, de Jong D, Van de Vijver MJ, Van't Veer LJ, Peterse JL (2008). Refinement of breast cancer classification by molecular characterization of histological special types. The Journal of pathology.

[18] Toikkanen S, Eerola E, Ekfors TO (1988). Pure and mixed mucinous breast carcinomas: DNA stemline and prognosis. Journal of clinical pathology.

[19] Matsukita S, Nomoto M, Kitajima S, Tanaka S, Goto M, Irimura T, Kim YS, Sato E, Yonezawa S (2003). Expression of mucins (MUC1, MUC2, MUC5AC and MUC6) in mucinous carcinoma of the breast: comparison with invasive ductal carcinoma. Histopathology.

[20] Park S, Koo J, Kim JH, Yang WI, Park BW, Lee KS (2010). Clinicopathological characteristics of mucinous carcinoma of the breast in Korea: comparison with invasive ductal carcinoma-not otherwise specified. Journal of Korean medical science.

[21] Bae SY, Choi MY, Cho DH, Lee JE, Nam SJ, Yang JH (2011). Mucinous carcinoma of the breast in comparison with invasive ductal carcinoma: clinicopathologic characteristics and prognosis. Journal of breast cancer.

[22] Chen L, Fan Y, Lang RG, Guo XJ, Sun YL, Cui LF, Liu FF, Wei J, Zhang XM, Fu L (2008). Breast carcinoma with micropapillary features: clinicopathologic study and long-term follow-up of 100 cases. International journal of surgical pathology.

[23] Liu F, Yang M, Li Z, Guo X, Lin Y, Lang R, Shen B, Pringle G, Zhang X, Fu L (2015). Invasive micropapillary mucinous carcinoma of the breast is associated with poor prognosis. Breast cancer research and treatment.

[24] Shet T, Chinoy R (2008). Presence of a micropapillary pattern in mucinous carcinomas of the breast and its impact on the clinical behavior. The breast journal.

[25] Ng WK (2002). Fine-needle aspiration cytology findings of an uncommon micropapillary variant of pure mucinous carcinoma of the breast: review of patients over an 8-year period. Cancer.

[26] Komenaka IK, El-Tamer MB, Troxel A, Hamele-Bena D, Joseph KA, Horowitz E, Ditkoff BA, Schnabel FR (2004). Pure mucinous carcinoma of the breast. American journal of surgery.

[27] Tseng HS, Lin C, Chan SE, Chien SY, Kuo SJ, Chen ST, Chang TW, Chen DR (2013). Pure mucinous carcinoma of the breast: clinicopathologic characteristics and long-term outcome among Taiwanese women. World journal of surgical oncology.

